# HIV Encephalopathy: pediatric case series description and insights from the clinic coalface

**DOI:** 10.1186/s12981-014-0042-7

**Published:** 2015-01-17

**Authors:** Kirsten A Donald, Kathleen G Walker, Tracy Kilborn, Henri Carrara, Nelleke G Langerak, Brian Eley, Jo M Wilmshurst

**Affiliations:** Division of Developmental Pediatrics, Department of Pediatrics and Child Health, University of Cape Town and Red Cross War Memorial Children’s Hospital, Cape Town, South Africa; Division of Pediatric Neurology, Department of Pediatrics and Child Health, University of Cape Town and Red Cross War Memorial Children’s Hospital, Cape Town, South Africa; Department of Pediatric Radiology, Red Cross War Memorial Children’s Hospital, Cape Town, South Africa; Department of Public Health and Family Medicine, University of Cape Town, Cape Town, South Africa; Department of Neurosurgery, University of Cape Town, Cape Town, South Africa; Division of Pediatric Infectious Diseases, Department of Pediatrics and Child Health, University of Cape Town and Red Cross War Memorial Children’s Hospital, Cape Town, South Africa

**Keywords:** HIV encephalopathy, Children, MRI, Brain, Developmental delay

## Abstract

**Background:**

The Human Immune Deficiency Virus (HIV) can manifest neurologically in both adults and children. Early invasion of the central nervous system by the virus, affecting the developing brain, is believed to result in the most common primary HIV-related neurological complication, HIV Encephalopathy (HIVE). In countries such as South Africa where many children have not been initiated on antiretroviral treatment early, HIVE remains a significant clinical problem.

**Methods:**

Children were selected from a clinic for children with neurologic complications of HIV, located at the Red Cross War Memorial Children’s Hospital, South Africa 2008–2012. Eligible subjects fulfilled the following inclusion criteria: aged 6 months-13 years; positive diagnosis of HIV infection, vertically infected and HIVE as defined by CDC criteria. Each participant was prospectively assessed by a Pediatric Neurologist using a standardized proforma which collated relevant details of background, clinical and immunological status.

**Results:**

The median age of the 87 children was 64 months (interquartile range 27–95 months). All except one child were on antiretroviral treatment, 45% had commenced treatment <12 months of age. Delayed early motor milestones were reported in 80% and delayed early speech in 75% of children in whom we had the information. Twenty percent had a history of one or more seizures and 41% had a history of behavior problems. Forty-eight percent had microcephaly and 63% a spastic diplegia. CD4 percentages followed a normal distribution with mean of 30.3% (SD 8.69). Viral loads were undetectable (<log 1.6) in 70% of the children. Brain imaging was performed on 56% with 71% of those imaged demonstrating at least one abnormality, most commonly white matter volume loss or signal abnormality.

**Conclusions:**

Amongst the cohort of children referred to this clinic, the diagnosis of HIVE was unrecognized in the general medical services, even in its most severe form. Developmental delay and school failure were major presenting problems. Co-morbidities are a frequent finding and should be sought actively in order to optimize management and promote best possible outcomes for this vulnerable group of children.

## Background

According to the World Health Organization, approximately 3.4 million children worldwide are suffering from Human immunodeficiency Virus (HIV) or Acquired immune deficiency syndrome (AIDS) with >90% of them residing in sub-Saharan Africa [1-3]. There are an estimated 300,000 HIV-infected children in South Africa not yet receiving antiretroviral therapy (ART), with an estimated 48,000 new infections among children annually [2,3]. The greatest proportion of these figures represent vertically acquired HIV, which includes infection in utero, at time of delivery and from breast milk [2].

The Western Cape, where the study was completed, commenced its public sector prevention of mother-to-child transmission (PMTCT) intervention programme in 2001. The initial PMTCT programme was based on the HIVNET 012 nevirapine (NVP) regimen, and provision of milk powder for all infants of mothers who elected to formula-feed their offspring for the first 6 months of life. Universal coverage of the PMTCT programme in the province was reached at the beginning of 2003. In mid-2004 an intensified PMTCT programme was introduced based on the CD4 count of all HIV-infected pregnant women. Those women with CD4 count ≤ 200 cells/μL were commenced on antiretroviral therapy (ART), while those with a CD4 count >200 cells/μL were commenced on zidovudine prophylaxis at 34–36 weeks gestation, and during labour administered a single dose of NVP. All infants born to HIV-infected mothers received a single dose of NVP at birth plus a 7-day course of AZT [4]. Between 2004 and 2013 this programme underwent several revisions including progressive liberalization of the criteria used to initiate ART in pregnant women, introduction of 6 week infant testing (previously 14 weeks), introduction of extended NVP prophylaxis for all HIV-exposed infants, promotion of breastfeeding, and the withdrawal of free-formula milk in 2012 [5].

HIV can have neurological manifestations in both adults and children. Early invasion of the central nervous system (CNS) by the virus, affecting the developing fetal and infant brain, is believed to result in the most common primary HIV-related CNS complication, HIV Encephalopathy (HIVE) [6]. In non-treated children prevalence reports have ranged from 20 to 60% [7-14]. HIVE refers to the disease, damage or malfunction of the brain caused by HIV-1. Static HIVE is an unchanging type of encephalopathy, whereas, progressive HIVE is associated with neuroregression. This complication can be present before significant immunosuppression has developed, however its presence in a child infected with HIV constitutes an AIDS-defining illness, [15] reflecting the severity of the disease. According to the Center for Disease Control (CDC), encephalopathy must include criteria in at least one of the following areas for at least 2 months in the absence of a concurrent illness: a) failure to attain or loss of developmental milestones or loss of intellectual ability, b) impaired brain growth or acquired microcephaly and/or c) acquired symmetric motor deficit [15].

While substantial numbers of children have benefitted from antiretroviral treatment (ART), significant barriers to early ART initiation continue to exist, compared to adults infected with HIV. This places untreated children at risk, particularly of central nervous system (CNS) sequelae. HIV-1 invades the developing CNS earlier and with greater severity than observed in adults [7-14]. In addition patients on ART may remain vulnerable to the effects of HIV on the brain because the CNS may be a reservoir for persistent viral replication [16].

Reported specific neurodevelopmental delays in young children associated with HIVE include delays in acquiring early motor and language skills [6,17,18]. These have included general gross motor skills and development co-ordination, muscle tone and deep tendon reflexes. In older children specific cognitive deficits, impaired motor skills and decreased language ability are also reported. Cognitive deficits include verbal and memory impairment and difficulty with visual-spatial integration and executive function [19,20].

Neuroimaging studies using more recently available quantitative magnetic resonance imaging (MRI) techniques are contributing to improved understanding of the underlying neurobiology of the primary effects of HIV on the developing brain. In a study by Ackermann *et al.*, the findings of white matter signal abnormalities in a significant proportion of the brains of young children (mean age: 31.4 months) initiated on ART under 3 months of age, may indicate that infiltration of the CNS by HIV occurs at an extremely early stage of infection [21]. These abnormalities are much more subtle than the global cerebral atrophy and or basal ganglia calcifications which are more typically described in the context of children with HIV encephalopathy (HIVE) [22]. In addition, two recent studies have reported subtle white matter microstructural abnormalities [23] and neurochemical abnormalities [24] in relatively well older children. In particular, Hoare *et al.* reported an association between poor performance on tests of executive function and attention with reduced white matter integrity in the corpus callosum in “slow-progressor” children who were considered too well to require ART. These white matter changes represent a variable combination of diffuse myelin loss, astroglial proliferation, and infiltration by mono and multinucleated macrophages [25]. Methods such as those described above, which can detect more subtle changes in especially white matter integrity, may be key in the investigation of both the timing and natural history of neurological complications of HIV. However, despite this emerging literature there remains a paucity of data from low and middle income countries (LMIC) where the utility of these investigations in a clinical context with high burden of disease, but limited resources and consequent different standard of care remains inadequately explored.

HIVE was found to be the most common primary neurological disorder of HIV infected children diagnosed in the HIV Neurology clinic at Red Cross War Memorial Children’s Hospital and in this report we aimed to describe this group of children in order to better understand their profile as well as identify challenges and opportunities for improved management in the clinical context.

## Methods

### Study population

This paper offers a descriptive profile of HIV infected children referred to a dedicated HIV-Neurology Clinic in Cape Town, South Africa and diagnosed with HIVE. The overall purpose of this clinic is to screen children identified by clinicians to be at risk for neurologic complications of HIV. Children are evaluated, investigated and a formal neurologic diagnosis given where appropriate. Following this there is a formulation of on-going care plans for these children. As part of the service a database has been collected to help understand the neurologic disease spectrum and demographics of our population group. The health system in South Africa is three-tiered such that the majority of children on ARTs receive treatment at a primary level, where access to neuro-imaging and neuro-cognitive testing is not available. Red Cross War Memorial Children’s Hospital is a tertiary level service with access to these facilities. Referrals to the HIV-Neuro clinic come from primary, secondary and tertiary levels. In addition to the specific neurologic conditions such as epilepsy and the complications of secondary CNS infections (primarily tuberculous and other bacterial meningitides), reasons for referral to this clinic include general concerns about developmental delay, particularly delay in onset of walking beyond 2 years, learning difficulties and behavior problems such as memory loss, hyperactivity, poor concentration and not referred as HIVE *per se*.

Children were selected from this clinic over the period 2008–2012. Eligible subjects were those who had attended the HIV Neurology clinic at least once and who fulfilled the following inclusion criteria: aged between 6 months and 13 years; positive diagnosis of HIV infection (includes initial and confirmatory tests), vertical transmission and HIVE as defined by CDC criteria. Children were excluded when they had a history of: an uncontrolled medical condition, such as the presence of an identified CNS condition (other than HIV), CNS infections e.g. Tuberculous Meningitis; drug or alcohol exposure in pregnancy; history of a head injury with loss of consciousness greater than 5 min or any radiological evidence of skull fracture; history of perinatal complications such as hypoxic ischemic encephalopathy or neonatal jaundice requiring exchange transfusion, or neurodevelopmental and or neuroregressive or genetic disorder not attributed to HIV.

### Data collection

Each participant was prospectively assessed by a medical specialist (KD or KW) using a standardized proforma which collated relevant details of background, clinical and immunological status information. Background information was collected from the care-giver at the time of assessment, though if supplementary information was available in the medical folder this was also included. Details included source of referral, age, sex, birth history (antenatal, birth complication, gestation and birth weight), past medical, early developmental history and a history of caregivers. In addition, care-givers were asked about the child’s home environment and schooling situation. This information was based on caregiver recall, although with respect to identifying age of achieving milestones a timeline follow-back approach was used to achieve best possible recall.

Clinical assessments performed included anthropometry (height, weight, head circumference (OFC)), full neurological and physical examination (indications of spastic diplegia and or long tract signs, microcephaly, peripheral neuropathy, visual and hearing impairment and child’s mood). In addition, immunological results collected consisted of most recent CD4 and viral load counts. Due to the disparate facilities from which the children had received their primary ART, information on immunological status at time of HIV diagnosis and ART commencement was available on too few children to include in the analysis.

Children selected for this study were also referred for either Computer Tomography (CT) or Magnetic resonance imaging (MRI) of the brain using the standard clinical neuroimaging protocol used at the hospital. These images were analyzed and reported by a Pediatric Neuroradiogist (TK) at Red Cross War Memorial Children’s Hospital. Informed consent is part of the protocol for MRI acquisition in young children. Due to the difficult socio-economic environments from which HIV infected children in South Africa generally come, as well as the high burden of medical appointments they are required to attend, failure to attend hospital appointments (particularly when the visit is not directly related to the management of HIV itself) is extremely common. This was the most common reason for children not having neuroimaging.

Collected information was anonymized and entered onto a password protected database in a private office. Ethical approval was obtained from the University of Cape Town Human Research Ethics committee (UCT HREC 126/2011).

### Statistical analysis

Background information, results of clinical assessments, immunological and imaging findings were analyzed by using Stata version 12 (Stata Corp LP, 4905 Lakeway Drive, College Station, Texas 77845 USA). All continuous data was assessed for normality using the Shapiro Wilk test, and parametric or non-parametric summary measures were reported as appropriate.

To determine if there was significant association between the age of starting ART and physical findings of microcephaly and spastic diplegia, the statistician compared the median age at ART commencement with two groups (positive or negative diagnosis) using the Wilcoxon rank-sum (Mann–Whitney test). This analysis was also repeated for testing the relationship between current immunological status and these physical findings.

In addition, analysis was done to determine whether there was an association between imaging abnormalities described in the cohort and the HIVE-defining physical findings of microcephaly and spastic diplegia, age at ART commencement and current immunological status. For each association prevalence ratios were estimated by using generalized linear regression.

Statistical significance level was set at p < 0.05.

## Results

### Study cohort background information

**Figure 1:**
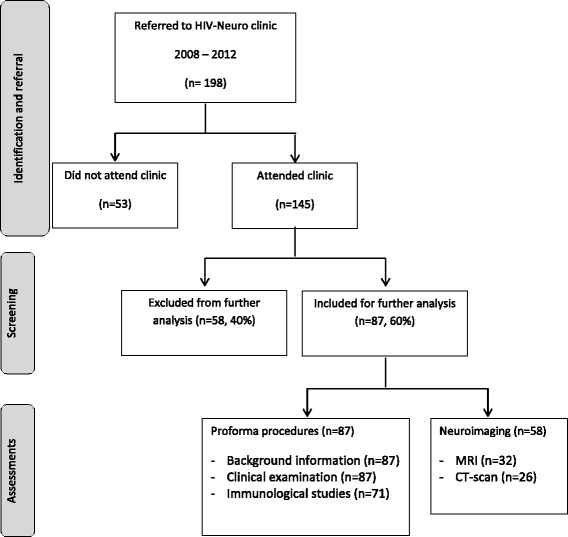
**Study algorithm.**

The algorithm as presented in Figure 1 provides an overview of the number of children identified, selected and included for proforma assessment and neuroimaging procedures. During the study period 145 children were seen in the HIV Neurology clinic, of which 87 children fulfilled the criteria for eligibility (HIVE) and were included in this study. The remaining 58 children had other neurological complications of HIV such as cerebro-vascular events, severe epilepsy or a background of other complicating medical factors (especially opportunistic CNS infections, perinatal complications and history of maternal substance use in pregnancy) which excluded an isolated diagnosis of HIVE. Only children who were diagnosed with HIVE were included in the analysis. Twenty nine of the 87 children were referred from primary health care clinics, 35 from secondary level hospitals and 23 from other clinics or wards at RCWMCH. Analysis of carer consistency showed 11 had consistent care with 2 parents, 38 had consistent care with one parent and 37 had had multiple carers during this time (which may have included a parent during some of that time).

Table 1 provides an overview of the background information of the HIVE study cohort. The median age of the children was 64 months interquartile range (IQR) of 27 to 95 months, with the majority aged between 2–9 years (87%). All except one child were on ART at time of first assessment, and 38 children (45%) had first received ART before the age of 12 months. Of the 87 children included, 85 were referred to the clinic as a result of concern regarding their failure to attain early developmental milestones, school failure or behavioral problems, but were not formally diagnosed with HIVE prior to being seen at the HIV-Neurology clinic.

**Table 1:** **Background information for children with HIVE**

**Variable (total number of assessments)**	**Subjects**	
	**n**	**%**
Gender (n = 87)		
Male	51	59
Female	36	41
Age group at examination (n = 87)		
<2 years	16	18
2 – 4 years	24	28
5 – 9 years	36	41
10 – 14 years	11	13
Age at ART commencement (n = 87)		
<12 months	38	44
12 – 36months	33	38
>36 months	14	16
Not reported	2	2
Developmental milestone walking (n = 75)		
On time	15	20
Delayed (>18 months)	60	80
Not old enough to tell	5	N/A
Not reported	7	N/A
Developmental milestone talking (n = 72)		
On time	18	25
Delayed (>18 months)	54	75
Not old enough to tell	4	N/A
Not reported	11	N/A
Home environment (n = 87)		
home	49	56
Institution, orphanage, foster home	13	15
bio foster (adopted by family member)	20	23
other foster	4	5
Not reported	1	1
School (n = 87)		
Mainstream pre-school	32	37
Mainstream school	35	40
Special school/care center	4	5
None	7	8
Not reported	9	10
Repeating grades		
Yes	37	43
No	27	31
Not reported	23	26

With regards to the early milestones, delayed walking was reported in 60/75 children (80%) and delayed early speech in 54/72 children (75%). Of the 81 children from whom a medical history was available 17 children (20%) had a history of one or more seizure and 14 (17%) had already been assigned a formal diagnosis of attention deficit hyperactivity disorder by their treating doctor when they were referred to the clinic. In addition, 33 children (41%) had a history of behavior problems as reported by their parents. Sixty-seven (86%) children were attending a mainstream educational facility at the time of assessment and more than half of the children of school-going age (57%) had had to repeat a school-year or was not age appropriate for their current school grade. All participants were of low socioeconomic status and 37 (43%) of them were living in a foster or adopted home environment.

### Clinical information

Anthropometric measures weight, height and head circumference were at least 2 standard deviations below that expected for age in more than half of the study-cohort. Of the 87 children who underwent physical exam, 42 children (48%) presented with microcephaly (head circumference <2standard deviations from the age norms on WHO growth charts), 55 (63%) with spastic diplegia. Thirty-three children (37%) had active ear disease (chronic otitis media). Clinical findings are detailed in Table 2.

**Table 2:** **Clinical findings for children with HIVE**

**Variable**	**Subjects**	
	**n**	**%**
Weight		
Normal	43	49
3^rd^ – 10^th^ centile	16	18
<3^rd^ centile	28	32
Height		
Normal	24	28
3^rd^ – 10^th^ centile	20	23
<3^rd^ centile	26	30
Not recorded	17	20
Physical exam		
Spastic diplegia/long tract signs	55	63
Microcephaly	42	48
Learning Difficulties	6	7
Peripheral Neuropathy	1	1
Visual impairment	1	1
Hearing impairment	2	2
Ears		
Normal	36	41
Acute otitis media	7	8
Chronic suppurative otitis media	26	30
not examined/recorded	16	18

### Immunological status

Recent CD4 count (percentage) and viral loads (log value) were available for 70 and 71 children with HIVE respectively. All the children about whom we had immunological information were on ART at the time of assessment. CD4 percentages followed a normal distribution with mean of 30.3% (SD 8.69). Viral loads were undetectable (<log 1.6) in 50 (70%) of the children. The mean viral load was log 2.07 (IQR 1.3-2.3).

### Neuroimaging

**Figure 2:**
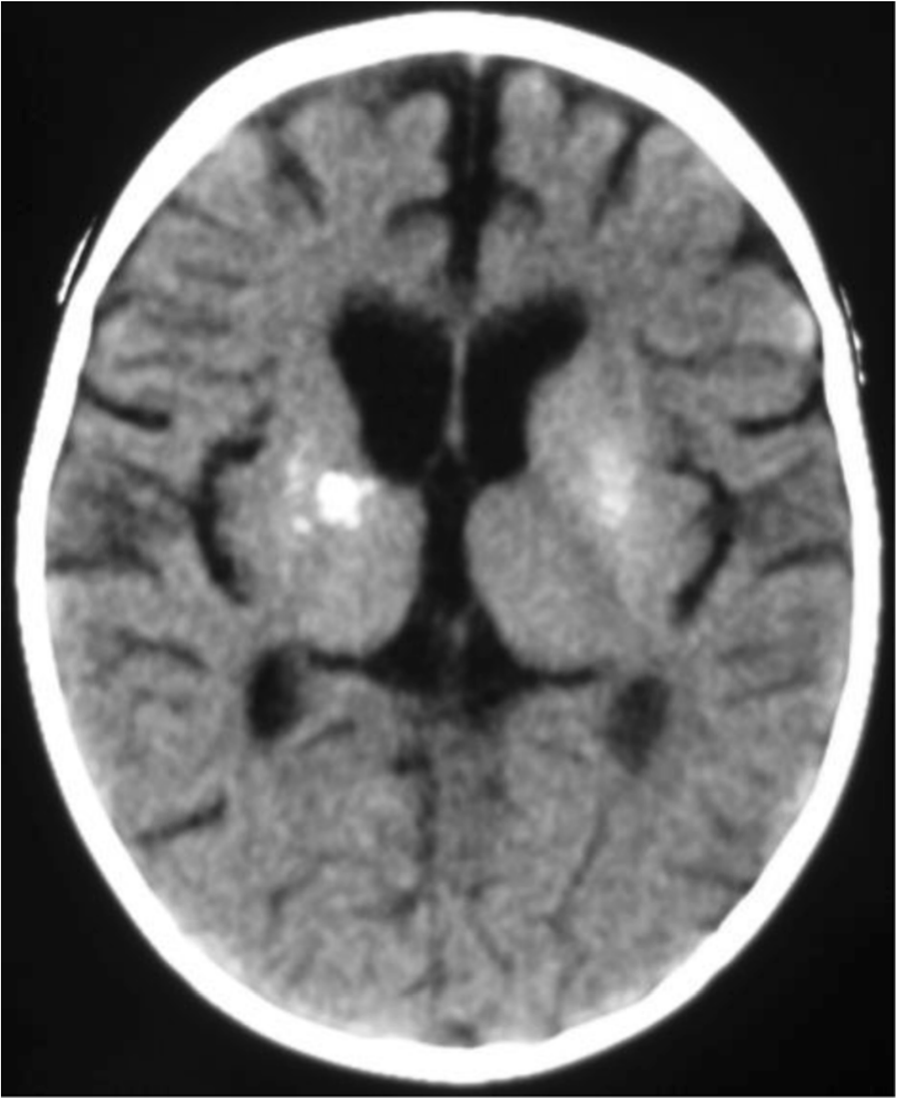
**Axial CT Brain showing bilateral basal ganglia calcification and cerebral shrinkage in a 1year old child with HIVE.**

**Figure 3:**
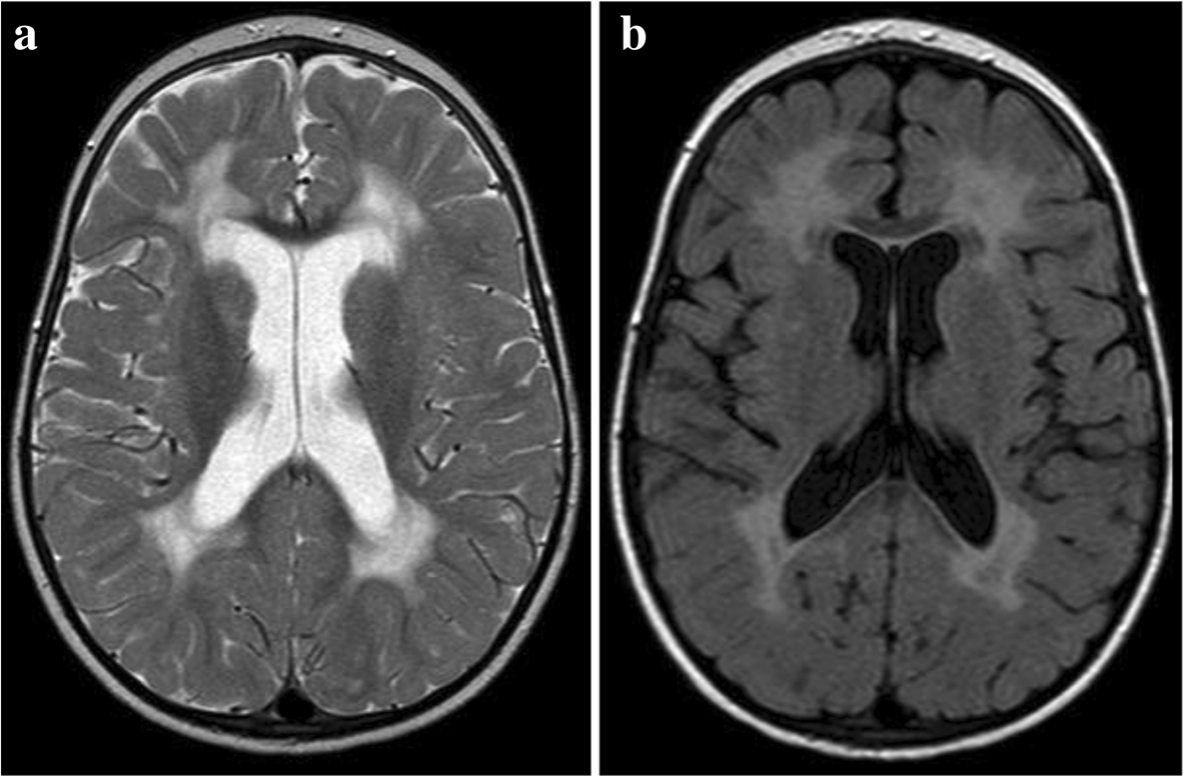
**Axial T2 (a) and FLAIR (b) images of an HIV-positive child showing bilateral symmetrical high signal in the periventricular white matter with normal basal ganglia.** Prominence of the cerebral sulci implies volume loss.

Brain imaging was successfully performed on 49 of the 87 (56%) children. Five children received both MRI and CT scans (their findings are aggregated) and 4 children failed their scan and so their findings are not reported, making up the total of 58 scans recorded in Figure 1. Fourteen children had normal scans, while 35 (71%) presented with at least one abnormality, six children with a combination of two abnormalities, two children with three abnormalities and three children with four abnormalities. Table 3 gives an overview of the CT and MRI imaging findings reported in the HIVE study-cohort and Figures 2 and 3 show examples of the most common neuroimaging findings.

**Table 3:** **Neuroimaging findings**

**Variable**	**Subjects**	
	**n**	**%**
Imaging		
Global Atrophy	10	20
Basal Ganglia Calcifications	4	8
White Matter Signal Change	9	18
White Matter Volume Loss	12	24
Grey Matter Volume Loss	4	8
Thinning of Corpus Callosum	9	18
Other	6	12
Normal	14	29

### Associations with microcephaly and spastic diplegia

There was no statistically significant difference in age at starting ART between those with and without microcephaly (p = 0.4, Wilkoxon rank-sum (Mann–Whitney test), z = −0.87). Similarly for spastic diplegia, there was no statistically significant difference in age at starting ART between those with and without spastic diplegia, although this approached significance (p = 0.06, Wilkoxon rank-sum (Mann–Whitney test), z = 1.83).There was no increased relative risk for a particular imaging abnormality with this group. Neither recent CD4 percentage values nor the presence/absence of detectable viral load correlated with the presence of particular neuroimaging findings in our cohort.

## Discussion

This study describes the clinical, immunological and imaging features of a clinical group of 87 children with HIVE attending a dedicated referral clinic for children with neurologic complications of HIV infection. Current figures clearly demonstrate the major burden of childhood HIV is carried by countries in sub-Saharan Africa. It is apparent that even in South Africa where the roll-out of ART to both HIV infected children and adults, has improved the outlook for children, HIVE remains a problem in clinical practice.

We established that amongst the cohort of children referred to us, the specific diagnosis of HIVE is frequently unrecognized in general medical services, even in its most severe form. Postulated reasons for this include busy clinics where the time taken to do a full neurology examination is difficult to find, or where clinicians, though recognizing the problem, referred the children for more detailed assessment in order to exclude other causes of the neurology problems in this group. Pediatric services and in particular more specialized neurology services are particularly under-represented across the African continent [26]. This implies that the milder forms are probably being missed in overburdened health systems such as that in South Africa and other resource-limited countries.

The reported prevalence of neurodevelopmental delays in HIV-infected children varies between 8 to >60% depending on the measures used and clinical populations [10,11,17,19,27,28]. Evidence is emerging that even children started on effective ART at a very young age, may demonstrate adverse neurologic outcomes in later childhood [18,20,21]. The presence of a history of delayed early motor and language milestones in the vast majority of this cohort is to be expected as their diagnosis pre-selects them into a severely affected group. However, this history of these delays suggest that the CNS damage in these children was present from early infancy and that despite almost uniformly good viral control at time of assessment, the residual effects of the virus on the developing neurological system is likely to be long-lasting.

Although with formal neuropsychological testing some of the children excluded from the diagnosis of HIVE in this cohort might have met criteria for the diagnosis, there will remain a group whose overall cognitive performance falls within the normal range (or within 2 SDs of the norm), but who have specific learning difficulties. Evidence for this spectrum of subtler neurocognitive effects is described in even completely asymptomatic children [23]. The concept of a “milder” form of neurocognitive disturbances in HIV infected children such as the adult condition of HIV-associated neurocognitive disorder (HAND) is recognized but is yet to achieve a consensus definition in children and adolescents. This condition may have significant effects in functional terms on the learning potential of the child (especially at school). This is a critical discourse, as it is likely to be important in informing treatment guidelines for these children especially in contexts where access to treatment may depend on clinical criteria. Although our older children did not consistently undergo formal neuropsychological testing, a significant proportion of these children were repeating grades at school. This can be regarded as a manifestation of functional neurocognitive compromise.

Co-morbidities were found to be significant management issues in this group of children with HIVE. Twenty percent had suffered one or more seizures, 40% had reported behavior problems with 18% a formal diagnosis of ADHD and 39% had active ear disease. Relatively “minor” conditions such as recurrent ear infections, which are inadequately managed, can affect a child’s hearing. Even mild hearing loss in this complex disorder where a child may have additional cognitive and or learning difficulties, could have serious long-term effects on their school outcome and future economic potential. High prevalence of behavior problems amongst other groups of children with chronic medical conditions is well described. HIV integration into the frontal cortex and connecting structures of the CNS are suggested as possible mechanisms for specific behavioral manifestations in this group [29,30]. The figure reported here is in keeping with some of the chronic physically disabling conditions such as Duchenne muscular dystrophy and other neuromuscular conditions (41%) where one may expect there to be a higher prevalence [31]. The high prevalence of co-morbidities in this group highlights the importance of actively enquiring after and managing these aspects of the child’s medical profile.

Although a definitive clinical or imaging predictor for HIVE has not been defined, risk factors associated with HIVE include maternal and child immune status, high CSF and plasma viral load, high circulating monocytes, timing of infection, route of transmission, and availability of early treatment [2,27,28]. This previously reported literature has implicated high viral loads as a predictor for HIVE. Over two thirds of our group had undetectable viral loads at time of assessment so this made it a largely unhelpful variable to use in our group. Information on peak viral loads or viral loads at the time of diagnosis prior to ART initiation may have been more helpful in this analysis. Of interest there was no statistically significant association between age at starting ART and the presence of microcephaly, though the association between age at starting ART and spastic diplegia approached significance in this group. Children manifesting spastic diplegia as their neurological criterion for HIVE are likely to represent the severe end of the spectrum of the disorder and the trend towards association with delayed ART in this group is consistent with studies which have demonstrated poorer neurological outcomes in children with deferred ART initiation [18].

Imaging findings described in HIVE have focused on a number of characteristics. These include Computer Tomography (CT) findings of generalized atrophy, ventriculomegaly, and basal ganglia calcification [32-35]. Although these are the most commonly reported signs of HIVE on CT scan; they appear not to be that common in absolute terms amongst children with HIVE and may represent the severe end of the spectrum of the disorder. Magnetic Resonance Imaging (MRI) studies have shown white matter microstructural changes amongst HIV-infected, but not encephalopathic children [23,35]. Neuroimaging changes in the brains of HIV-infected children could represent important indicators of disease. A study showed correlations between changes in the corpus callosum and significant reduced executive function and attention tests, as well as relations between changes in superior longitudinal fasiculus and reduced functional performance [23]. On the other hand, in another older study, 76% of asymptomatic HIV-positive children were found to have at least one abnormality of global atrophy as well as more specific white matter changes on brain CT scan [32]. Very few of these studies have correlated the neuroimaging findings with neurological findings in the children they describe. In our cohort, clinical neuroimaging sequences, even in an experienced specialist unit did not correlate usefully with physical findings. A large proportion of scans (29%) were completely normal despite all the children having abnormal neurological findings suggestive of upper motor neuron pathology (microcephaly and or spastic diplegia). It is probable that more sophisticated MRI techniques such as diffusion tensor imaging would be able to identify abnormalities in white matter integrity at a microstructural level, however this is not likely to represent a practical clinical approach in our setting in the foreseeable future.

Introduction of ART is associated with prolonged survival rates of children suffering from HIV by promoting a broad spectrum of general and specific health measures including normal growth and development, improved immunity, reduced risk of and vulnerability to infections [36,37]. Its essential role in the effective management of the disease is now undisputed. ART is also associated with dramatically decreased the prevalence of HIVE in the United States from 35-50% to less than 2% [2,38]. Furthermore, early initiation of ART results in improved neurodevelopmental outcomes compared to deferred initiation [18]. However, in resource-limited contexts such as South Africa, where ART is not always easily accessible and treatment is frequently delayed beyond infancy, HIVE remains an important and frequently undiagnosed clinical problem.

Limitations of our study include relatively small sample size and cross-sectional study design. All data was captured from a data-base of a working clinic at a tertiary centre in South Africa. The vast majority of HIV infected children are primarily treated at primary level health institutions which are staffed by medical officers and not pediatricians or sub-specialists in neurology, child psychiatry or developmental pediatrics and where the burden of attending to the immediate life-threatening medical issues are likely to result in only the severe developmental or behavioral issues being identified. As a result, the relatively small number of children in this report is likely to represent superficial evidence of a much larger under-recognized problem. Another significant limitation includes the fact that behavioral history and psychosocial information was collected via carer report rather than standardized testing and represents the reality of what even a specialized clinic is able to offer in the clinical setting in the present environment. However, both clinicians who assessed the children are experienced in addressing this scope of problem and the time-line follow-back method was used to optimize parental recall where appropriate. In addition, the lack of formal neurocognitive evaluations on the older age group of children presenting to the clinic means that the diagnosis of HIVE was made on one of the two physical CDC criteria. As such this group of 87 children most likely represents the more severe end of the HIVE spectrum (i.e. all the children with a diagnosis of HIVE had an abnormal neurological examination, making analysis for clinical predictors very difficult). The lack of useful clinical associations between physical findings and immunological or imaging findings is not entirely surprising given the cross-sectional study design and particular cohort.

## Conclusion

The assessment of this group of children revealed some useful insights for clinical practice. Regular anthropometric measurements including height and head circumference should be recorded in order to identify children at risk of growth failure as well as making the diagnosis of HIVE where appropriate. Structural neuroimaging may not be helpful in the day-to-day management of a significant group of children with HIVE. It is however, useful in excluding secondary conditions where indicated, especially where long tract signs are present such as spastic diplegia. School failure is a major problem in this group and in a society where HIV remains highly stigmatizing and the school is frequently not aware of the child’s diagnosis, many parents are reluctant to seek help for their child’s learning problems. Co-morbidities are a frequent finding in the South African setting and need to be sought actively in order to optimize management and promote best possible educational outcomes for this vulnerable group of children.

Recently circulated national guidelines for the initiation of ART in South Africa now recommends blanket treatment for the <60 month age group (with older children needing to meet clinical or immunological criteria). However, until this year, official national policy only recommended immediate treatment for infants under 12 months of age [39]. As a result there is still a large pool of children who would not have been included in the blanket ART initiation group and who remain untreated and vulnerable to the effects of the virus on the developing central nervous system. This study highlights that in order to improve the quality of life of these children earlier diagnosis of neurological risk at primary health care levels is needed.
